# Genetic Divergence and Connectivity among Gene Pools of *Polyprion americanus*

**DOI:** 10.3390/ani13020302

**Published:** 2023-01-15

**Authors:** Pablo Presa, Alfonso Pita, Nédia R. Matusse, Montse Pérez

**Affiliations:** 1 Laboratory of Marine Genetic Resources (ReXenMar), Centro de Investigación Mariña, Universidade de Vigo, 36310 Vigo, Spain; 2 Sustainable Marine Aquaculture and Biotechnology Research Group (AquaCOV), Centro Oceanográfico de Vigo, Instituto Español de Oceanografía-CSIC, 36390 Vigo, Spain

**Keywords:** connectivity pattern, genetic structure, hybrid species, migration ecology, *Polyprion* spp., wreckfish

## Abstract

**Simple Summary:**

The wreckfish *Polyprion americanus* is a long-living grouper distributed anti-tropically. Three regional gene pools have been described so far in this species, i.e., the Atlantic North, the Atlantic Southwest, and the Indo-Pacific Ocean. This study addresses the interspecific divergence within the genus *Polyprion* spp. as well as the intrapopulation structure of *P. americanus* from the Atlantic North, by analyzing mitochondrial DNA and nuclear DNA gene markers on a comprehensive sampling effort. A highly divergent gene pool from South Africa was conspicuously intermediate between *P. americanus* and *P. oxygeneios*, which suggests its putative hybrid origin between those species. The inclusion of the South Africa pool produced a very high nuclear DNA divergence among *Polyprion* spp. populations which contrasts with the large genetic homogeneity of the Atlantic North stock. Inferred significant migration rates suggest a longitudinal connectivity pattern which strengthens the bi-directional migratory hypothesis in the Atlantic North gene pool.

**Abstract:**

Three regional gene pools of *Polyprion americanus* have been described so far, i.e., the North Atlantic, the Southwest Atlantic, and the Indo-Pacific Ocean. However, there is taxonomic uncertainty about the Southeast Atlantic population and there is suspicion on the existence of a third species of *Polyprion* in that area. Additionally, prior studies have shown a lack of genetic structuring in the Atlantic North. Nonetheless, a more conspicuous characterization of intensity, periodicity, and direction of migration are needed to properly understand the wreckfish connectivity pattern in the North Atlantic population. This study addresses the interspecific concerns highlighted above as well as the intrapopulation structure of *P. americanus* from the Atlantic North, using the mitochondrial DNA Cytochrome Oxidase I gene and nuclear DNA microsatellite markers on a comprehensive sampling effort. The highly divergent gene pool from South Africa was characterized by the specific Mitochondrial DNA *PamCOI.Saf* haplotype. Its molecular composition and phylogenetic status were conspicuously intermediate between *P. americanus* and *P. oxygeneios*, which suggests its putative hybrid origin between those species. Microsatellite variation exhibited a high differentiation (24%) among four putative *Polyprion* spp. gene pools which contrasts with the large genetic homogeneity within the Atlantic North stock (*F*_SC_ = 0.002). The significant migration rates inferred upon Bayesian algorithms suggest a longitudinal bi-directional connectivity pattern which strengthens the migratory hypothesis previously suggested on demographic data in the Atlantic North gene pool.

## 1. Introduction

The wreckfish *Polyprion americanus* (Bloch and Schneider 1801) is a pan-oceanic species distributed in both hemispheres and excluding the tropics [1]. In the Northern Hemisphere, *P. americanus* inhabits both sides of the Atlantic Ocean, the Mid-Atlantic ridge, and the Atlantic Archipelagos (Bermuda, Azores, Madeira, and Canaries) as well as the Mediterranean Sea [2]. In the Southern Hemisphere, this species inhabits the Atlantic West (Brazil and Argentina), the Middle Atlantic (Tristan da Cunha Islands, Gough Island), and the Atlantic Southeast (Vema Seamount and South Africa) [3,4,5]. It has also been described in the South Indian Ocean (St. Paul and Amsterdam Islands) as well as in the Pacific South (Southern Australia and New Zealand), where it coexists with the congeneric species *P. oxygeneios* [2]. The wreckfish is a long-lived gonochoristic teleost (78 years for the females and 58 years for the males) which reaches ~2 m in length and 100 kg in weight, e.g., [6]. It exhibits low mortality in the wild (M = 0.14 per year for combined sexes) as well as a high growth rate (k = 0.03–0.08 per year for combined sexes), e.g., [7]. Males accomplish maturity around 11 years and ≈70 cm in length [8] and females mature around 14 years and ≈84 cm in length [9] from February to March in the Blake Plateau/Charleston Bump off the U.S. Atlantic coast [8,10] at rocky bottom depths ranging 450–850 m [11]. Fecundity in the Atlantic Northwest population ranges 1.4–4.1 million pelagic eggs from females of 933–1280 mm in length [8]. Pelagic juveniles up to ~2–3 years (~60 cm) drift with surface currents [12,13] and are found near floating objects before they recruit to the bottom to initiate the adult demersal phase [2,9,11].

The significant genetic divergence observed between samples of *P. americanus* from both hemispheres using mtDNA suggested that latitudinal migration across the tropics was improbable [14]. Indeed, a further microsatellite study identified three well differentiated gene pools, i.e., the Atlantic North and the Mediterranean Sea, the Atlantic South (Brazil), and the Pacific South (Australia and New Zealand) [1]. A longitudinal migration was suggested upon the dispersal ability of pelagic wreckfish as coupled with circulation patterns within hemispheres e.g., [15]. For instance, evidence exists on the northwards spawning migration of this species along the Southeastern American coast [16] or from Australia to New Zealand in late winter [17]. The low abundance of juvenile wreckfish in the Atlantic Northwest led to hypothesize that pelagic juveniles drifted in a Northeastern direction with the Gulf Stream, approached Atlantic Northeast Archipelagos [14], and returned to Blake Plateau in about 9–11 months [10,18]. Genetic studies using PCR-RFLPs profiles of the ND1 mitochondrial DNA gene [14] as well as microsatellites [1] came to reinforce the above hypothesis.

In the last three decades, there has been an increasing interest on the wreckfish fishery in both Atlantic coasts e.g., [19] that was motivated by its good flesh quality, large size, and high market price. The high growth rate exhibited during its pelagic stage coupled with the ease of its domestication, have also contributed to rising interest in its aquaculture development [20,21,22]. Such industrial interest was parallel to the high fishing pressure that finally brought about much concern on the sustainability of this fishery on both sides of the Atlantic e.g., [18]. The wreckfish fishery from the Atlantic Southwest is critically endangered in the Brazilian coasts which stock has been included in the IUCN red list [9,15,23] and landings have been diminishing by 80% since 2000 in Argentina. The wreckfish fishery of the Atlantic Northeast has been mainly exploited by Spanish and Portuguese fleets which landings peaked in 2007 but soon returned to catches observed at the beginning of the last decade. Additionally, the Mediterranean landings peaked in 2004 and despite that they had been traditionally low, they have shown a frank decrease in the last decade [24]. The wreckfish fishery from the Atlantic Northwest is no longer viable in Bermuda e.g., [2] while its catches and CPUE grew rapidly in the USA on the unregulated fishery (1987–1989) and TAC quotas enforced thereafter became rapidly exhausted [7]; the ITQ system implemented in 1992, which enforced fishing flexibility as well as the closure of the fishery during the main annual spawning period, have decreased fishing capacity, improved market value of the catch, and conserved fish stocks and habitats [25]; this stock is not subject to overfishing based on 2020 catch data (https://www.fisheries.noaa.gov/species/wreckfish, accessed on 4 November 2022). 

Management of the Atlantic North wreckfish in a sustainable manner requires evidence of the population dynamics of this species, i.e., either eastern and western populations are fully isolated from each other or form a panmictic population characterized by a consistent pattern of gene flow across the Atlantic. The first goal of this study was to gain knowledge on some phylogenetic gaps within *Polyprion* sp. using the Cytochrome Oxidase I gene and the Rhodopsin gene, by including samples of the congeneric species *P. oxygeneios* which overlaps with the former in the Southern Hemisphere. Early records of *Polyprion* sp. from South Africa were assigned to *P. americanus* [3,4,5], but later it was suggested that they may correspond to *P. oxygeneios* [26]. Preliminary mtDNA profiles and microsatellite genotypes allowed it to be hypothesized that a third species of *Polyprion* might exist in the Indian Ocean waters off South Africa [1]. Therefore, the systematics of *Polyprion* sp. From South Africa also need to be clarified to prevent overharvesting of a cryptic *Polyprion* species in a single fishery. The second objective focused on evaluating the consistency of a single gene pool of *P. americanus* in the Atlantic North [1,14] by inferring migration rates afforded from microsatellite variation on a comprehensive collection of samples from the USA and Europe.

## 2. Materials and Methods

### 2.1. Sampling and DNA Extraction

**Figure 1:**
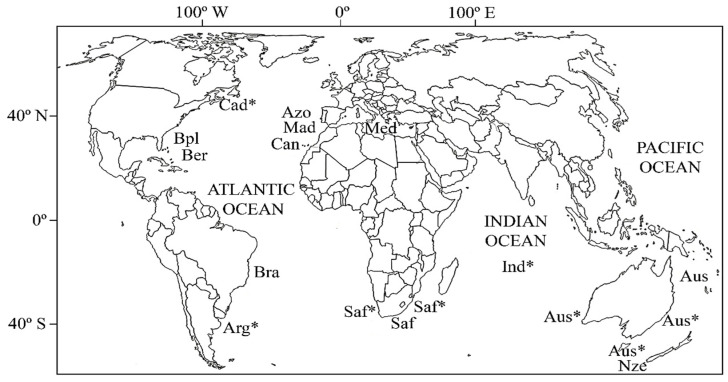
Sampled regions of *Polyprion americanus*: Arg*, Mar de Plata (Argentina); Aus, Northwestern Australia; Aus*, Australia (from east to west: New South Wales, Tasmania and Western Australia); Azo, Azores; Ber, Bermuda; Bra, Brazil; Bpl, Blake Plateau; Cad*, Canada; Can, Canary Islands; Ind*, South Indian Sea; Mad, Madeira; Med, Mediterranean Sea; Nze, New Zealand; Saf, South Africa; Saf*, South Africa (Atlantic ocean and Indian Ocean). Asterisks indicate samples which COI sequences were retrieved from BOLD database.

A total of 581 specimens of *P. americanus* were collected during research campaigns on the species range carried out in the last 20 years (Table 1; Figure 1); 452 out of 581 specimens were sampled by the Department of Natural Resources (Marine Resources Research Institute, Hollings Marine Laboratory, USA) and muscle samples were preserved in buffer 1% Sarcosyl-Urea until DNA was purified using the phenol–chloroform method [27]. The number of specimens from the Azorean Archipelago was increased with 50 fin-tissues collected in 2012 and the sample from the Canarias Archipelago consisted of 79 muscle tissues collected in 2013 (Table 1). The morphological identification of the samples was performed upon catch by researchers from the collaborator institutions (see the Acknowledgements section). Tissues were preserved in pure ethanol until DNA extraction using the method FENOSALT [28]. Total DNA was resuspended in 50 µL of 1xTE buffer and its quality and quantity were determined using a NanoDrop-1000 spectrophotometer v.3.7 (THERMOFISHER SCIENTIFIC, Waltham, MA, USA). DNA integrity was checked after electrophoresis in 1% agarose gels and purified DNA was kept at −20 °C until PCR amplification and sequencing.

### 2.2. Amplification of DNA Sequences

The nuclear DNA Rhodopsin gene and a fragment of the mitochondrial DNA Cytochrome Oxidase I gene (COI) employed in species identification e.g., [29] were used to explore the homogeneity of gene pools within *P. americanus* as well as to infer the phylogenetic relationships within *Polyprion* spp. In order to complete and compare current molecular data, we used additional sequences of both genes from this species as retrieved from the Barcode of Life Database (BOLD, http://www.boldsystems.org; accessed on 3 December 2014) and from the National Center for Biotechnology Information (NCBI, http://www.ncbi.nlm.nih.gov/; accessed on 6 July 2014). An 800 bp fragment of the Rhodopsin gene was PCR amplified with primer pair Rh193F/Rh1039R [30] on the genomic DNA of forty-eight individuals from nine samples. A 660 bp fragment of COI gene was PCR amplified with primer pair FishF2/FishR2 [31] on the genomic DNA of 41 individuals from 15 samples. The PCR reaction for both genes consisted of 15 μL containing 1xNH4 Reaction Buffer (BIOLINE), 0.2 mM of each dNTP, 1.5 U BioTaq DNA polymerase (BIOLINE), 0.15 µM of each primer, 10–40 ng of DNA template, and 1.5 mM of MgCl_2_. Amplification conditions for both genes consisted of an initial denaturing step at 95 °C for 10 min, followed by 35 cycles at 95 °C for 45 s, 55 °C (COI) or 50 °C (Rhodopsin) for 1 min, and 72 °C for 1 min, ending with a final extension at 72 °C for 10 min. Amplicons were purified with Exonuclease I and Alkaline Phosphatase following the manufacturer’s instructions (ThermoFisher Scientific, Waltham, MA, USA) and sequenced at CACTI facilities (Scientific and Technological Research Assistance Centre, University of Vigo, Vigo, Spain) using the PCR primers.

### 2.3. Molecular Divergence among Lineages

The G+C content of COI sequences, haplotype diversity, nucleotide diversity per site, average number of nucleotide differences between sequences, and Fu’s *F*s neutrality statistics were calculated with DNAsp v. 5.0 [32]. Nucleotide diversity (*P*_i_) within the major lineages of COI sequences (*P. americanus*, *P. oxygeneios*, and *Polyprion* sp. from South Africa) was calculated with MEGA V6.0 [33]. The molecular divergence between COI lineages was assessed using the average number of nucleotide substitutions per site between lineages (*D*_xy_) and the number of net nucleotide substitutions per site between lineages (*D*_a_) [34] using DNAsp. A maximum-parsimony network of COI haplotypes was constructed with the median joining algorithm [35] as implemented in NETWORK 4.6.1 [36] using default settings. Recombination rate per gene [37] and the minimum number of recombination events [38] within COI sequences from the three lineages were obtained with DNAsp. The divergence time between species was inferred using a standard mtDNA-clock calibrated among 26 pairs of major intraspecific fish phylogroups [39]. Dating back the putative hybridization event between *P. americanus* and *P. oxygeneios* which could have given rise to *Polyprion* sp. from South Africa employed the average clock-pace of 2% per million years (Myr). Calculation of the average number of nucleotide substitutions per site between present-day lineages was performed after [39] as,

*D*_(pam−pox)_ = *D*_xy_ − 1/2 (*P*_i(pam)_ + *P*_i(pox)_) (1)

*D*_((pam&pox)−saf)_ = (*D*_(pam−saf (obs))_ + *D*_(pox−saf (obs))_) − 1/2 (*D*_(pam−pox)_) (2)

where *D*_xy_ is the absolute nucleotide divergence between *P. americanus* and *P. oxygeneios*, *P*_i_ is the nucleotide diversity within species, *D*_(pam−pox)_ is the net nucleotide divergence between *P. americanus* and *P. oxygeneios*, *D*_(pam−saf (obs))_ and *D*_(pox−saf (obs))_ are the uncorrected average number of nucleotide substitutions per site between the species considered and *Polyprion* sp. from South Africa, and *D*_((pam&pox)−saf)_ is the average nucleotide divergence between *P. americanus*-*P. oxygeneios* and *Polyprion* sp. from South Africa.

### 2.4. Phylogenetic Inference

A total of 73 high-quality sequences were obtained as 37 of COI and 36 of Rhodopsin (Table 1), edited with BIOEDIT 7.2.5 (Isis Pharmaceuticals Inc. ©1997–2004) and aligned using CLUSTAL W [40] from a BIOEDIT subdirectory. The DNA sequences were assessed in the BLAST tool against GENBANK databases to confirm their ascription to *Polyprion* spp. Nine COI sequences of *P. americanus* and six COI sequences of *P. oxygeneios* from BOLD database [41] were included in the phylogenetic analysis. Rhodopsin sequences were co-analyzed with four sequences of *P. americanus* and one sequence of *P. oxygeneios* retrieved from GENBANK [42] (Table 1). Transition/transversion ratio and overall disparity index of sequences were calculated with MEGA V6.0 [33]. The best substitution model was chosen upon the Akaike Information Criterion (AIC) implemented in jMODELTEST [43] as available in PHYLEMON 2.0 [44]. Initial trees for the heuristic search were obtained with the algorithms neighbor-joining and BioNJ on a matrix of pairwise distances from the maximum composite likelihood approach (MCL), and the subsequent selection of the topology was performed after the highest log-likelihood value. Maximum likelihood phylogenetic trees (ML) were inferred for both, the mtDNA gene (COI) and the nuclear DNA gene (Rhodopsin), using MEGA. Robustness of the tree nodes was estimated using 5000 bootstrap replicates [45]. A neighbor-joining tree [46] was built with the recombination detection program–RDP4 [47] using 10,000 bootstrap replicates and the JC distance model [48] to explore the relationships among COI haplotypes from *P. americanus* and *P. oxygeneios*.

### 2.5. Amplification of Microsatellite Markers

Two PCR duplexes were worked out to assess microsatellite variation in this species. Duplex I comprised microsatellites PamD1 and PamA5 [22]. Duplex II comprised microsatellites Pam006 and Pam021 [1]. PCR reactions for both duplexes were carried out in a final volume of 15 μL containing 1xNH_4_ reaction buffer (670 m M Tris–HCl, pH 8.8, 160 mM (NH_4_)_2_SO_4_, 100 mM KCl, 0.1% Stabilizer (BIOLINE), 0.2 mM of each dNTP, 0.75U BioTaq DNA polymerase (BIOLINE), 10 ng of DNA template, and a MgCl_2_ concentration of 1.7 mM (duplex I) and 3 mM (duplex II)). PCR primers were used at 0.27 µM each for PamD1, 0.33 µM each for PamA5 (duplex I), and 0.3 µM for duplex II. PCR amplifications of both duplexes were carried out in a Mastercycler Gradient Thermocycler (EPPENDORF, Hamburg, Germany) and consisted of an initial denaturing step at 96 °C for 10 min, followed by 30 cycles at 94 °C for 30 s, 58 °C (duplex I) or 55 °C (duplex II) for 1 min, and 72 °C for 1 min, with a final extension at 72 °C for 10 min. An aliquot of the amplified products was electrophoresed in 2% agarose gels to assess the expected amplification size and quality. One microliter of each amplicon was mixed with 10.75 μL of Hi-Di formamide and 0.25 μL of Genescan500 ROX size-standard and run in an ABI Prism-3130 Genetic Analyzer (APPLIED BIOSYSTEMS®, Waltham, MA, USA) from CACTI. In order to minimize genotyping errors, ABI genotypes were called independently by three researchers using the software Genemarker V1.97 (SOFTGENETICS LLC, State College, PA, USA).

### 2.6. Data Analysis of Microsatellite Variation

Allele frequencies, number of alleles (*A*), allelic richness (*R*_S_), and fixation indexes [49] were calculated with FSTAT 2.9.3.2 [50]. Test of putative null alleles was performed with FREENA [51] using 1000 permutations. The probability associated to *F*_IS_ was generated with the Markov chain method implemented in GENEPOP 4.2.1 [52] using 20 batches of 5000 iterations each. The observed heterozygosity (*H*_O_) and the expected heterozygosity (*H*_E_) were calculated with GENEPOP. The differentiation index *D*_EST_ [53] and its statistical significance among samples were calculated upon 1000 bootstrap replicates using DEMETICS 0.8-5 [54]. Correction for multiple tests was performed using the false discovery rate approach [55]. The relationship among samples upon variance components was visualized in a bi-dimensional space using a principal coordinates analysis (PCoA) as implemented in GENALEX 6.5 [56]. The number of gene pools (k) was inferred with BAPS 6 [57] using the approximate sampling coordinates, a spatial mixture analysis [58], and an admixture analysis based on the mixture clustering of 100,000 Bayesian iterations [59]. The k-value was also assessed through 2,000,000 Bayesian iterations under the spatial model [60] and the uncorrelated allele frequency model [61] implemented in GENELAND 4.0.0 [62]. The statistical power of the microsatellite dataset to detect population structure was tested with POWSIM [63]. Per-locus AMOVA as implemented in ARLEQUIN 3.5 [64] was used to split hierarchically the genetic variance of the whole dataset among the main clusters recovered with BAPS/GENELAND and PCoA. Nominal statistical levels for fixation indexes *F*_CT_ and *F*_SC_ were determined after 1,023 permutations. Post-migration rates (m) between pairs of samples were inferred after the Bayesian multilocus genotypic method implemented in BayesAss V3.0 [65] and consisted of 5,000,000 MCMC iterations, a 1,000,000 burn-in threshold, and a 1,000-iteration sampling interval. A priori settings of mixing parameters were Δ*M* = 0.95, Δ*A* = 0.95, and Δ*F* = 0.95. Final acceptance rates for proposed changes after convergence were Δ*M* = 0.54, Δ*A* = 0.72, and Δ*F* = 0.87. Pairwise pre-migration rates were estimated with the Bayesian algorithm implemented in BIMr [66] as a complementary test on migration trends, because of the uncertain accuracy of m-values from BayesAss in low *F*_ST_ scenarios. Priors were settled after 20 initial pilot runs of 20,000 iterations each, followed by 5 MCMC independent runs of 110,000 iterations each, a burn-in of 10,000 iterations, and a thinning interval of 50 iterations. Migration estimates were taken from the run with the lowest Bayesian deviance [66]. Samples relationships using microsatellite variation were assessed with PHYLIP 3.696 [67] using the neighbor-joining method on the Cavalli-Sforza chord genetic distance on 10,000 bootstrap replicates of the allele frequencies. Gene frequencies of the outgroup species (*P. oxygeneios*) were taken from [1].

## 3. Results

### 3.1. Haplotypic Diversity and Molecular Divergence

A total of 36,800 bp sequences of Rhodopsin co-aligned with those from databases (Accession numbers: gi133923802, gi129561557, gi129561555, gi133923804, gi393007797) produced a final dataset of 450 nucleotides containing 440 conserved sites, 10 singletons, and no parsimonious informative sites. Haplotype diversity was Hd = 0.172 from five haplotypes. The overall average disparity index was zero and the number of base substitutions per site averaged over sequence pairs was 0.001 ± 0.000. The best substitution model following Akaike criterion was HKY+G [68] and the best ML tree (log-likelihood = −688.278) showed a full polytomy comprising all sequences from *P. americanus* and *P. oxygeneios*. No further genetic analyses were performed on the Rhodopsin gene due to its highly conserved non-informative sequence. 

**Figure 2:**
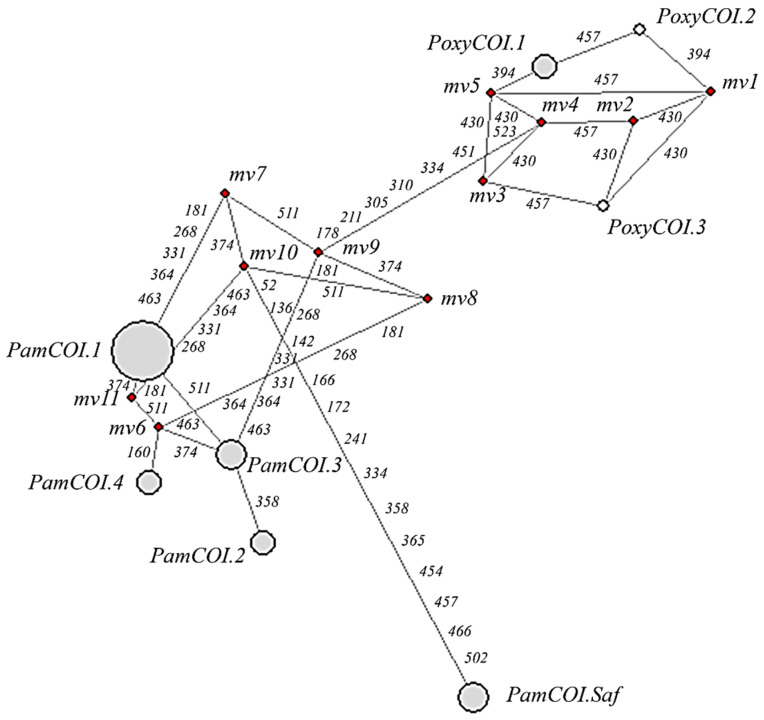
Median-joining network showing the mutational distance among mitochondrial DNA COI haplotypes from *P. americanus* (*PamCOI*) and *P. oxygeneios* (*PoxyCOI*) (see Table 2 above for sequence data). Median vectors (mv, in red dots) indicate likely extant but non-sampled sequences. The diameter of circles indicates the relative frequency of each haplotype.

The G+C content of the COI gene was 47.9%, and the number of segregating sites was S = 31 out of 32 variable sites. Haplotype diversity was Hd = 0.719 from h = 8 haplotypes, and the global nucleotide diversity (per site) was P_i_ = 0.0145. The average number of nucleotide differences was k = 7.402 and the Fs test of Fu was significant for all variable sites within *Polyprion* spp. as expected among species (Fs = 6.673; *p* = 0.034). Fs was non-significant among sequences of *P. americanus* (Fs = 0.613; *p* = 0.711) suggesting mutation-drift equilibrium in this species. The most common haplotype of *P. americanus* (*PamCOI.1*) was observed in the Atlantic North, haplotypes *PamCOI.2* and *PamCOI.3* in the Atlantic South, *PamCOI.4* in the Indian Ocean, Eastern Australia, and New Zealand, and *PamCOI.Saf* in South Africa (Table 2). Haplotype *PoxyCOI.1* was observed in the Indian Ocean and in Eastern and Western Australia, *PoxyCOI.2* in Eastern Australia, and *PoxyCOI.3* in Western Australia (Table 2). The net evolutionary divergence and the average number of nucleotide substitutions per site between *P. americanus* and *P. oxygeneios* were one-third less than the divergence of those species with *Polyprion* spp. from South Africa (Table 3). The mutational relationships plotted in the haplotypic network (Figure 2) showed 1-2 steps divergence among haplotypes either within *P. americanus* or within *P. oxygeneios*. The mutational divergence between those species comprised 15 changes and their separation with the South Africa sample comprised 20 changes. The recombination rate estimated for the COI sequence was 0.001 upon analysis of 52 sequences. The minimum number of significant recombination events was three, i.e., between the COI nucleotide positions 364 and 374, 451 and 457, 457 and 523 (see Table 2). The net (corrected) nucleotide divergence between *P. americanus* and *P. oxygeneios* was 0.026055 and the average nucleotide divergence (corrected) between present day (*P. americanus*–*P. oxygeneios*) and *Polyprion* spp. from South Africa was 0.0276175, which dates it back to 1,380,875 year bp using a COI average divergence rate of 2% per Myr in fishes.

### 3.2. Phylogenetic Inference

**Figure 3:**
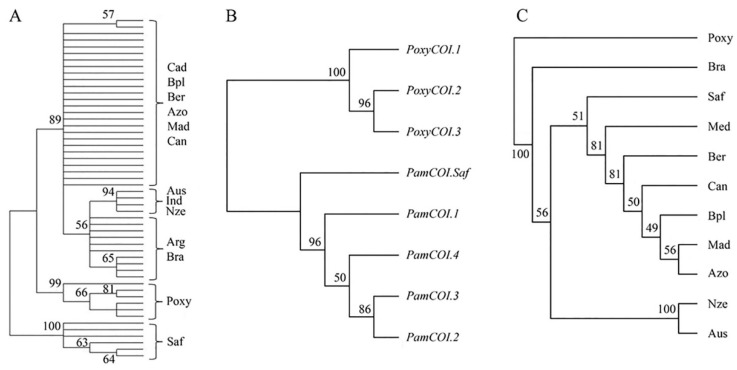
(**A**) Maximum likelihood phylogenetic tree on COI sequences (log-likelihood −1109.4004) implemented with the HKY+G model of substitution. The percentage of trees in which the associated taxa clustered together is shown next to the nodes. Bootstrap values less than 50% are not shown; (**B**) Neighbor-joining dendrogram built with eight COI haplotypes from *P. americanus* and *P. oxygeneios*. Percentages of trees with the same clustering out of 10,000 resampled trees are shown on nodes. Bootstrap values less than 50% are not shown; (**C**) Neighbor-joining dendrogram based on the chord distance from the allele frequencies of microsatellites. Percentages of trees with the same clustering out of 10,000 resampled trees are shown on nodes.

The phylogenetic analysis comprised 53 COI sequences aligned in a final matrix of 613 nucleotides, as 37 sequences from current samples and 16 ones retrieved from databases (Table 1 and Table 2). The overall average disparity index between COI sequences was 0.001 and the estimated transition/transversion ratio was 4.19. The phylogenetic tree inferred with the ML method used the HKY+G model and a discrete Gamma distribution (gamma = 0.050) to model differences of evolutionary rate among sites. The phylogenetic tree with the highest log-likelihood (−1109.4) comprised three well-supported clades (Figure 3A). The major clade comprised samples of *P. americanus* from the Atlantic North (Cad, Bpl, Ber, Azo, Mad, and Can), South America (Bra and Arg), and an internal subclade comprising the South Indian Ocean (Ind) and Oceania (Nze and Aus) samples. The second well-supported clade comprised all samples of *P. oxygeneios* and a third clade comprised all samples from South Africa (Saf). The NJ tree built on eight COI haplotypes showed two main supported clades, one comprising the three haplotypes of *P. oxygeneios* (*PoxyCOI.1*, 2, 3) and the other one comprising the four haplotypes of *P. americanus* (*PamCOI.1*, 2, 3, 4) (Figure 3B). Within *P. americanus*, a supported subclade was formed by haplotypes *PamCOI.2*, 3 from the Atlantic South (Bra and Arg) which was divergent from the rest of haplotypes from the Atlantic North and Oceania (*PamCOI.1*,4). The haplotype *PamCOI.Saf* formed an intermediate clade between the two major clades of *P. americanus* and *P. oxygeneios*.

### 3.3. Microsatellite Variation

**Figure 4:**
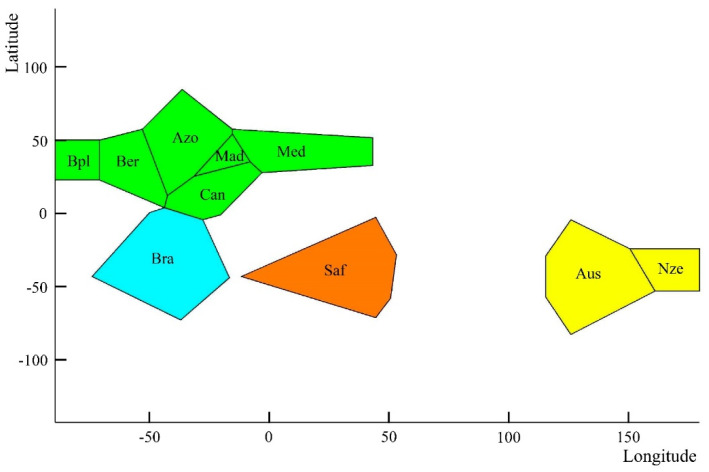
Spatial mixture clustering analysis performed by BAPS on GPS coordinates (latitude and longitude) of each sample were set according to fishing records. Different colors indicate different gene pools as recovered with BAPS.

The number of alleles per locus ranged between 14 (locus PamD1) and 25 (locus Pam006). The four microsatellites were polymorphic in all samples except marker PamD1 in the Mediterranean (Figure S1). The putative frequencies of null alleles were generally below 0.10 except seven cases over that figure but not ascribed to a specific locus or to a population. Modal alleles were distinct among samples, e.g., marker PamD1 showed a modal size of 171 bp in the Atlantic North but 175 bp in Oceania (Figure S1). Samples from Bermuda (Ber), Madeira (Mad), Mediterranean Sea (Med), South Africa (Saf), Australia (Aus), and New Zealand (Nze) were in Hardy–Weinberg equilibrium (HWE) in all markers, while samples from Black Plateau (Bpl), Azores Islands (Azo), and Brazil (Bra) showed heterozygote deficit in some loci (Table S1). The most likely number of gene pools retrieved from the Bayesian admixture analysis of BAPS (k = 1 to 10) was k = 4 with probability *p* = 0.9997 (Figure 4), i.e., (1) North Atlantic samples and Mediterranean ones (Bpl, Ber, Mad, Azo, Can, Med), (2) Brazil (Bra), (3) South Africa (Saf), and (4) Oceania (Aus and Nze). The spatial model implemented in GENELAND produced the same gene pool scenario as BAPS (data not shown). The microsatellite dataset showed a statistical power of 1.0 using both, ten independent populations sampled worldwide (*F*_ST_ = 0.117) and four gene pools recovered by GENELAND (*F*_ST_ = 0.241). No statistical power was observed within Atlantic populations (*F*_ST_ = 0.001) across simulations. The global *F*_ST_ among samples was significant (*F*_ST_ = 0.121; *p* = 0.001) but it was non-significant among samples from the Atlantic North (*F*_ST_ = 0.002) (Table 4). The variation among groups was significant in the AMOVA levels enforced using the sample pools identified with BAPS (k = 4; *F*_CT_ = 0.241) and PCoA/GenAlEx (k = 5; *F*_CT_ = 0.235) (Table 4). The differentiation parameters (*F*_ST_ and *D*_EST_) were not significantly different from zero in pairwise comparisons within groups (i.e., *F*_ST_ ranged 0.000–0.005 among Atlantic North samples as well as between Australia and New Zealand (*F*_ST_ = 0.020) (Table 5)). Both indexes were highly significant in pairwise comparisons between regions, e.g., *D*_EST_ ranged 0.605–0.782 between Oceania and the North Atlantic group (Table 5). Significant Bayesian-inferred migration rates [65] were observed eastwards in the Atlantic North, i.e., from Blake Plateau grounds (Bpl) to the rest of North Atlantic grounds, e.g., to Azores (Azo, m = 0.258 ± 0.045), Madeira (Mad, m = 0.182 ± 0.089), Canaries (Can, m = 0.153 ± 0.093), and the Mediterranean Sea (Med, m = 0.101 ± 0.091) (Table S2). A significant migration rate was also observed in the westward direction from Azores to Blake Plateau (m = 0.124 ± 0.101) as well as from Australia (Aus) to New Zealand (Nze) (m = 0.174 ± 0.070). Significant m-rates were also recovered between Northeast Atlantic samples and the Northwest Atlantic (Bermuda) using the Bayesian algorithm that assumes post-fecundation but pre-migration rates [66]. That algorithm also identified a significant connectivity among Atlantic Northeast Archipelagos (Table S3). The NJ dendrogram built from microsatellite allele frequencies supported a major clade comprising all samples of *P. americanus* including the South Africa ones (Figure 3C).

## 4. Discussion

### 4.1. Haplotype Diversity and Phylogenetic Inference on COI

The low molecular divergence among COI sequences within the species *P. americanus* and *P. oxygeneios* indicates that all the samples belong unambiguously to the specific mitochondrial lineage of those species. This result agrees with the synonymies worked out on 20 nominal species of *Polyprion* [69] but is at odds with the suggestion that *P. moeone* and *P. oxygeneios* were the only valid species occurring in Australia and New Zealand e.g., [70]. The divergence between the Atlantic North and the Atlantic Southwest in the COI gene was not as strong as reported for the mitochondrial gene ND1 [14]. Particularly, the samples from the latter region (Brazilian and Argentinian) cluster intermingled as expected from the reported northward displacement to Southern Brazil in winter and spring and back to Argentina in summer and autumn [15]. Additionally, the support of a single COI subclade for samples from the South Pacific and the Indian Ocean is congruent with the migration reported between Australia and New Zealand (see also [1,14]) as well as with the connectivity inferred between wreckfish from the Indian Ocean and Western Australia using demographic metrics [71]. The haplotype heterogeneity observed within *P. oxygeneios* reflects the regional divergence of gene pools, i.e., haplotypes *PoxyCOI.2* (East Australia) and *PoxyCOI.3* (West Australia) from haplotype *PoxyCOI.1* (Indian Ocean and East and West Australia). However, such variation in *P. oxygeneios* does not have systematic value and likely represents a limited connectivity among temporal spawning stocks, such as that inferred with microsatellites between the South Island of New Zealand and other regional samples [72]. Such low divergence among haplotypes of *P. oxygeneios* is in the range observed among haplotypes of *P. americanus* (2–3 substitutions), i.e., far less than the variation observed between species (15 substitutions between *P. americanus* and *P. oxygeneios* or 20 substitutions between these latter and the *PamCOI.Saf* haplotype from South Africa). 

Additionally, a conspicuous phylogenetic separation was patent among specific COI haplotypes within species, i.e., *PamCOI.1* in the North Atlantic, *PamCOI.2,3* in the South Atlantic, *PamCOI.4* in the Indo-Pacific region, and *PamCOI.Saf* in South Africa. 

### 4.2. The South Africa Wreckfish

The current NJ dendrogram on microsatellite variation is largely consistent with previous UPGMA reconstruction [1]. However, the adhesion of the South Africa sample to the *P. americanus* cluster contrasts with its position outside *P. americanus* and *P. oxygeneios* in the COI phylogenies. This intergenomic conflict suggests that wreckfish from South Africa are a distinct mitochondrial lineage within *Polyprion* spp. that bears a good deal of nuclear DNA from *P. americanus*. While the nuclear DNA ascription of the Saf sample to *P. americanus* could be due to homoplasy, the strong support of the *PamCOI.Saf* haplotype between those of *P. americanus* and *P. oxygeneios* suggests the putative hybrid origin of the South Africa wreckfish. Indeed, the molecular homogeneity of COI sequences within *P. americanus* and within *P. oxygeneios* contrasts with the large molecular divergence of those species with the *PamCOI.Saf* haplotype. This result is consistent with the highly distinctive mtDNA and microsatellite profiles previously observed on *Polyprion*-like specimens from South Africa [1]. Based on a conventional molecular clock of 2% divergence among COI sequences per Myr (0.69–3.00% molecular fork in fishes, [39]), the South Africa *Polyprion* divergence from its putative parental species would date back to 1.4 Myr bp (0.92–4.00 Myr). Such a temporal fork comprises the advent of glacial cycles and cold-water upwelling around South Africa some 2.5 Myr ago [73], a time when some species such as trumpet fishes in the East Atlantic were isolated from the Indian Ocean [74].

### 4.3. Microsatellite Variation among Regional Populations of P. americanus

The disjoint allelic distributions and the divergence of the modal allele size of all microsatellites between regions, confirm the regional divergence within *P. americanus* already observed with mtDNA COI sequences. The heterozygote deficit of two microsatellites in the samples from Blake Plateau (Bpl), Azores Islands (Azo), and Brazil (Bra) can be due to interannual fluctuation of allele frequencies in Bpl (five years) and Azo (three years) as well as by the genetic divergence of Brazil (Bra) regarding the Atlantic North population where microsatellites where isolated from [1,22]. Despite the Mediterranean sample clustered to the Atlantic North pool using Bayesian computation, no firm conclusions can be made on its genetic status. Its apparent fixation for allele 171 of locus PamD1 could either be due sampling drift or to migration drift from the Atlantic into the Mediterranean trough the Gibraltar Strait (see next subsection), such as reported in other marine fishes e.g., [75]. The absence of significant cross-equatorial migration rates between the Atlantic North and Brazil, South Africa, or the Pacific South, as well as between these latter, is in agreement with the substantial separation reported among those gene pools [1]. However, while some records of *P. americanus* suggest that Australian and New Zealand stocks could belong to separate species [9], the significant migration rate between Australia and New Zealand is congruent with their genetic ascription to *P. americanus* e.g., [1].

Three out of four Bayesian gene pools observed in *P. americanus*, i.e., Atlantic North, Brazil, and the South Pacific (Australia and New Zealand) are congruent with previous studies on mtDNA [14] and microsatellites [1]. However, the South Africa sample appears as a fourth gene pool with a high *D*_EST_ divergence from the rest of gene pools, as suggested upon mtDNA COI variation. The estimates of gene flow (*F*_ST_) and differentiation (*D*_EST_) were congruent with each other (positively correlated, data not shown) especially at low divergence levels, i.e., within the Atlantic North and within the Indo-Pacific. However, *D*_EST_ was 2-3 fold higher than *F*_ST_ at higher differentiation levels where the effect of distinct allelic composition among regional pools was more informative than heterozygosity-based *F*_ST_ differences [53,76].

### 4.4. Microsatellite Variation in P. americanus from the Atlantic North

Range values of parameters *F*_ST_ and *D*_EST_, confirm that the Atlantic North is a spatiotemporal genetically homogeneous stock unit and the Indo-Pacific is the most divergent gene pool within wreckfish. Assuming expected fluctuations in range and number of alleles due to sample sizes, number of markers, and spatiotemporal variation of samples, a good congruence is observed on population genetic metrics in wreckfish from the Atlantic North between studies. For instance, current observations of thirty-five alleles from four microsatellites in one hundred and forty-five Azorean specimens is congruent with observation of forty-six alleles from six microsatellites in one hundred and eighteen specimens [1] or thirty-eight alleles from five microsatellites in forty specimens [22]. Congruence also exists with previous studies on wreckfish from wider Atlantic sampling efforts (e.g., [1], see its values subsequently within parenthesis) as for instance in the range of alleles per locus of 7–19 (6–19) and expected heterozygosity 0.333–0.830 (0.480–0.831) in four (six) microsatellites and four hundred and seventy-one (three hundred and thirty-seven) specimens from six (seven) samples collected in the North Atlantic and the Mediterranean Sea. 

The single gene pool formed by fifteen Atlantic samples as inferred with Bayesian approaches and differentiation coefficients (e.g. *F*_ST_ = 0.002) as well as with RFLPs on 1.5 kb PCR amplicon from the ND1 mtDNA gene [14] or with six microsatellites in ten Atlantic North samples (*F*_ST_ = 0.0004) [1] point to the existence of a single-unstructured wreckfish population in the Atlantic North as occurs in other Atlantic fishes such as *Gadus morhua* [77]. Spatial genetic homogeneity requires intermittent gene flow, and the characterization of exchange patterns requires knowledge on intensity, temporality, and direction of migration episodes [78]. In wreckfish, [1] hypothesized that juveniles found in the Atlantic Northeast originated in part from spawning in the Blake Plateau and were transported across the Atlantic by ocean currents [14,79]. However, [80] using parasites and [14] using RFLPs on the ND1 mitochondrial gene suggested that spawning could also occur in the Azores and in the Mid-Atlantic Ridge. In this regard, the Azores Current, the Canary Current, and the North-Subtropical Gyre [81,82] could carry offspring spawned in Azores to Madeira and Canaries and perhaps to Bermuda and Blake Plateau. Noteworthy, the eastward migration from Blake Plateau to the Atlantic Northeast, the way back to Blake Plateau, and the inter-archipelago connection, are congruent with m-rates inferred with BayesAss and BIMr. The differences between those algorithms regarding the direction connecting the same samples reside in the assumptions of the exchange model, i.e., post-migration rates (BayesAss) or post-fecundation but pre-migration rates (BIMr). Surface flow across the Gibraltar Strait could carry pelagic juvenile wreckfish into the Mediterranean Sea [81] where some spawning activity could also exist [12]. Such an Atlantic–Mediterranean connection is shown herein by the genetic proximity among all samples from the Northern Hemisphere as well as by current m-rates into the Mediterranean (BayesAss).

## 5. Conclusions

In addition to the three known regional gene pools within *P. americanus*, i.e., the North Atlantic, the South Atlantic, and the Indo-Pacific, a new highly divergent gene pool from South Africa is characterized by the specific mitochondrial DNA *PamCOI.Saf* haplotype. This haplotype places the wreckfish sample from South Africa at an intermediate phylogenetic position between *P. americanus* and *P. oxygeneios* which suggests its putative hybrid origin. The taxonomic recognition of the South Africa wreckfish as a different species within *Polyprion* spp. deserves more morphological and genetic investigation.

Genetic differentiation levels, Bayesian clustering inferences, exchange rates, and phylogenetic consistency among markers and methods showed that *P. americanus* forms a single metapopulation in the whole Atlantic North and should be taken as a single management unit. Such unit conforms a spatiotemporal gene pool on which joint USA–EU management efforts should be implemented to boost optimization and sustainability of this fishery.

## Figures and Tables

**Table 1:** Characteristics of samples from *P. americanus* analyzed in this study (see Figure 1 for sample location). Code, regional code for sample locations; N_MIC_, number of individuals genotyped with microsatellites; N_COI_, number of individuals with information for the Cytochrome Oxidase I sequence (COI); N_Rod_, number of individuals with information for the Rhodopsin gene sequence (Rod).

Regional Origin	Sampling Year	Code	N_MIC_	N_COI_	N_Rod_
Atlantic North					
Azores	1993, 1996, 1997, 1998, 2012	Azo	145	5	1
Bermuda	1996, 1997	Ber	13	2	3
Blake Plateau	1995, 1996, 1997	Bpl	200	6	5
Canada	Unreported	Cad *	0	1	0
Canary Islands	2013	Can	79	9	10 ^b^
Madeira	1993, 1996, 1997	Mad	28	3	7 ^b^
Mediterranean Sea	1994	Med	6	0	0
Atlantic South					
Argentine	2008	Arg *	0	3	0
Brazil	1995	Bra	82	7	7
West South Africa	2010	Saf *	0	1	0
Pacific South					
East Australia	1995	Aus	12	0	2
Southeast Australia	1994, 1995, 1998	Aus *	0	2(3) ^a^	0
New Zealand	1995	Nze	9	1	3(1) ^c^
Indian Ocean					
Western Australia	1995	Aus *	0	0(2) ^a^	0
South Indian Ocean	Unreported	Ind *	0	1(1) ^a^	0
East South Africa	1997	Saf	7	4	2
East South Africa	2010	Saf *	0	2	0
Total			581	53	41

* Asterisks on sample codes indicate samples which COI sequences were taken from the database BOLD (https://www.boldsystems.org/index.php/Public_BINSearch?searchtype=records; accessed on 3 December 2014). ^a^ Samples comprising specimens from both, *P. americanus* and *P. oxygeneios* (in parenthesis). ^b^ Samples comprising sequences of *P. americanus* from NCBI (accessed on 6 July 2014), as two from Madeira (GenBank Accessions EF439297.1 and EF439298.1) and two from Canarias (GenBank Accessions EF427494 and EF427493.1). ^c^ The New Zealand sample is a specimen of *P. oxygeneios* from NCBI (GenBank Accession JX04917).

**Table 2:** Nucleotide polymorphisms, absolute (Freq), and relative (%) frequency of eight COI haplotypes from *Polyprion* spp. and their distribution per region (see codes in Table 1). Haplotypes *PamCOI* and *PoxyCOI* were observed in *P. americanus* and *P. oxygeneios*, respectively. Sequence entries from databases cited as footnotes were used in the reconstruction of the ML phylogenetic tree. Current COI haplotypes were used to build the NJ-tree reconstruction.

	**Nucleotide Position**																																												
		1	1	1	1	1	1	1	2	2	2	3	3	3	3	3	3	3	3	3	3	3	4	4	4	4	4	4	5	5	5														
	5	3	4	6	6	7	7	8	1	4	6	0	0	0	1	3	3	5	6	6	7	9	3	5	5	5	6	6	0	1	2		Absolute haplotype frequency per region												
Haplotype	2	6	2	0	6	2	8	1	1	1	8	4	5	7	0	1	4	8	4	5	4	4	0	1	4	7	3	6	2	1	3	Freq (%)	Bpl	Cad ^a^	Ber	Azo	Mad	Can	Bra	Arg ^b^	Saf	Saf ^c^	Ind ^d^	Aus ^e^	Nze
*PamCOI.1*	C	C	G	T	A	C	T	C	A	C	G	T	T	A	A	A	A	T	T	T	G	C	G	G	C	C	C	G	T	A	T	26 (50.0)	6	1	2	5	3	9							
*PamCOI.2*	.	.	.	.	.	.	.	.	.	.	.	.	.	.	.	.	.	C	.	.	.	.	.	.	.	.	.	.	.	G	.	4 (7.7)							3	1					
*PamCOI.3*	.	.	.	.	.	.	.	.	.	.	.	.	.	.	.	.	.	.	.	.	.	.	.	.	.	.	.	.	.	G	.	6 (11.5)							4	2					
*PamCOI.4*	.	.	.	C	.	.	.	.	.	.	.	.	.	.	.	.	.	.	.	.	A	.	.	.	.	.	.	.	.	G	.	4 (7.8)											1	2	1
*PamCOI.Saf*	T	T	A	.	G	T	.	A	.	A	A	C	.	G	.	G	.	.	C	C	A	.	.	.	T	T	T	A	C	.	.	6 (11.5)									4	2			
*PoxyCOI.1*	.	.	.	.	.	.	C	A	G	.	A	.	C	.	G	G	C	.	C	.	.	.	A	A	.	T	T	.	.	.	C	4 (7.7)											1	3	
*PoxyCOI.2*	.	.	.	.	.	.	C	A	G	.	A	.	C	.	G	G	C	.	C	.	.	T	C	A	.	T	T	.	.	.	C	1 (1.9)												1	
*PoxyCOI.3*	.	.	.	.	.	.	C	A	G	.	A	.	C	.	G	G	C	.	C	.	.	T	C	A	.	.	T	.	.	.	C	1 (1.9)												1	

^a^ BOLD sample SCFAC569-06 from Canada (GenBank accession KC015825); ^b^ BOLD samples FARG621-09 (*PamCOI.2*), FARG620-09 (*PamCOI.3*), and FARG622-09 (*PamCOI.3*) from Mar del Plata, Argentina (Robert Hanner, Biodiversity Institute of Ontario, 2009); ^c^ BOLD samples DSFSG406-10 (GenBank accession HQ945983, Dirk Steinke, Biodiversity Institute of Ontario, 2011) and DSLAG1796-12 (GenBank accession KF489705, D. Steinke, A.D. Connell and T.S. Zemlak, Biodiversity Institute of Ontario, 2013) from South Africa; ^d^ BOLD samples ANGBF7784-12 (*P. americanus*; GenBank accession AB639846) and ANGBF7812-12 (*P. oxygeneios*; GenBank accession AB639853) (T. Yanagimoto and K. Hoshino, National Research Institute of Fisheries Science, 2011). ^e^ BOLD samples FOA595-04 and FOA596-04 of *P. americanus* from New South Wales (Australia). BOLD Samples FOA597-04 (*PoxyCOI.2*) and FOA600-04 (*PoxyCOI.1*) from Tasmania (East Australia), FOA598-04 (*PoxyCOI.3*) and FOA599-04 (*PoxyCOI.1*) from Western Australia, and FOA601-04 (*PoxyCOI.1*) from New South Wales (East Australia) belong to *P. oxygeneios* (GenBank accession DQ107914, DQ107915, DQ107900, DQ107903, DQ107901, DQ107902, and DQ107904, respectively, [31]).

**Table 3:** Nucleotide diversity (*P_i_* ± *SD*) within three lineages of COI sequences (on the diagonal). Estimates of net evolutionary divergence between COI lineages (*d* ± *SD*, above the diagonal). Average number of nucleotide substitutions per site between COI lineages (*D_xy_*, below the diagonal).

COI Lineages	*P. americanus*	South Africa	*P. oxygeneios*
*P. americanus* ^a^	0.00196 ± 0.0011	0.093 ± 0.056	0.062 ± 0.042
South Africa ^b^	0.03799	0.000 ± 0.000	0.104 ± 0.067
*P. oxygeneios* ^c^	0.02838	0.04330	0.00269 ± 0.0019

^a^ This group included seven COI entries of *P. americanus* from BOLD database: Canada (*n* = 1), Argentine (*n* = 3), South Indian Ocean (*n* = 1), and Southeastern Australia (*n* = 2) (see Table 1 and Table 2, and Figure 1). ^b^ This group included two COI sequences of “*P. americanus*” from South Africa (BOLD database). ^c^ This group included five COI sequences of *P. oxygeneios* from Australia and one more from the South Indian Sea (BOLD database).

**Table 4:** Hierarchical AMOVA on the variation of microsatellites in *P. americanus* from both Hemispheres. Asterisks indicate the probability that observed values were equal or smaller than those expected by chance, * *p* ≤ 0.01; ns: non-significant.

Hierarchical Level	Source of Variation	Sum of Squares	Variance Component	% Variation	Fixation Index
Whole dataset (ten locations, nineteen samples)	Among populations	143.445	0.17484	12.07	*F*_ST_ = 0.121 *
	Within populations	1257.935	1.27385	87.93	
Atlantic North samples ^a^ (six locations, fifteen samples)	Among populations	7.479	0.00294	0.24	*F*_ST_ = 0.002 ^ns^
	Within populations	979.253	1.21576	99.76	
	Among populations within groups	9.023	0.00282	0.17	*F*_SC_ = 0.002 ^ns^
	Within populations	1245.518	1.27101	74.94	*F*_ST_ = 0.251 *
BAPS groups ^b^ (*k* = 4 gene pools)	Among groups	134.422	0.40514	24.09	*F*_CT_ = 0.241 *
	Among populations within groups	9.023	0.00279	0.17	*F*_SC_ = 0.002 ^ns^
	Within populations	1257.935	1.27385	75.74	*F*_ST_ = 0.243 *
PCoA groups ^c^ (five gene pools)	Among groups	135.630	0.39196	23.49	*F*_CT_ = 0.235 *
	Among populations within groups	7.815	0.00305	0.18	*F*_SC_ = 0.002 ^ns^
	Within populations	1257.935	1.27385	76.33	*F*_ST_ = 0.237 *

^a^ This sample group comprises the Mediterranean Sea sample. ^b^ Four BAPS groups comprising samples from [Atlantic North and Mediterranean], [Bra], [Saf], [Aus and Nze]. ^c^ Five PCoA groups comprising samples from [Atlantic North], [Mediterranean], [Bra], [Saf], and [Aus and Nze].

**Table 5:** Pairwise estimates of differentiation (*D*_EST_, below diagonal) and fixation index (*F*_ST_, above diagonal) between wreckfish samples (codes in Table 1). Significance of both estimates were corrected for multiple tests with the FDR algorithm of [55]; * *p* ≤ 0.001; NA, test not feasible due to the low number of genotyped specimens in samples Med and Saf.

	Bpl	Ber	Azo	Mad	Can	Med	Saf	Bra	Aus	Nze
**Bpl**	-	0.000	0.002	0.001	0.005 *	0.000 ^NA^	0.083 ^NA^	0.226 *	0.340 *	0.314 *
**Ber**	0.000	-	0.000	0.000	0.000	0.000 ^NA^	0.059 ^NA^	0.193 *	0.333 *	0.317 *
**Azo**	0.004	0.000	-	0.000	0.005 *	0.000 ^NA^	0.089 ^NA^	0.219 *	0.327 *	0.303 *
**Mad**	0.000	0.000	0.000	-	0.000	0.000 ^NA^	0.103 ^NA^	0.217 *	0.366 *	0.348 *
**Can**	0.006	0.000	0.005	0.000	-	0.000 ^NA^	0.074 ^NA^	0.201 *	0.317 *	0.293 *
**Med**	0.176 ^NA^	0.193 ^NA^	0.187 *	0.206 ^NA^	0.205 *	-	0.141 ^NA^	0.253 ^NA^	0.514 ^NA^	0.468 ^NA^
**Saf**	0.146 ^NA^	0.167 *	0.188 *	0.190 *	0.169 *	0.238 ^NA^	-	0.081 ^NA^	0.304 ^NA^	0.264 ^NA^
**Bra**	0.655 *	0.702 *	0.673 *	0.695 *	0.652 *	0.640 *	0.472 *	-	0.221 *	0.204 *
**Aus**	0.706 *	0.649 *	0.703 *	0.736 *	0.705 *	0.782 ^NA^	0.772 *	0.710 *	-	0.020
**Nze**	0.650 *	0.605 s*	0.652 *	0.698 *	0.649 *	0.670 *	0.694 *	0.717 *	0.021	-

## Data Availability

Data is contained within the article or Supplementary Material.

## Supplementary Materials

The following supporting information can be downloaded at: https://www.mdpi.com/article/10.3390/ani13020302/s1, Figure S1. Distribution of allele frequencies (in bp) of four microsatellites in *Polyprion americanus* as broken per sampling region (sample codes in Table 1); Table S1. Genetic parameters of four microsatellites in ten sampling areas of *Polyprion americanus* (sample codes in Table 1); Table S2. Migration rate *m* (± 95% CI) between wreckfish samples (codes in Table 1) from the first row (Donors) to those in the first column (Receptors) as inferred with BayesAss. Bolded figures indicate migration rates significantly different from zero; *m*-values on diagonal cells correspond to within-sample migration rates; Table S3. Migration rate *m* (±95% CI) between wreckfish samples (codes in Table 1) from the first row (Donors) to those in the first column (Receptors) as inferred with BIMr. Bolded figures indicate migration rates significantly different from zero; *m*-values on the diagonal correspond to within-sample migration rates.
