# Supplementary material for: Genetic Divergence and Connectivity among Gene Pools of Polyprion americanus
Source: Animals (Basel). 2023 Jan 15;13(2):302. doi: 10.3390/ani13020302 (PMC9854721; doi:10.3390/ani13020302)
Supplement: Supplementary file 1 [file animals-13-00302-s001.zip › animals-2132177-SI.pdf]

animals

MDPI

Article

Genetic Divergence and Connectivity among Gene Pools of *Polyprion americanus*

Pablo Presa, Alfonso Pita, Nedia R. Matusse and Montse Pérez

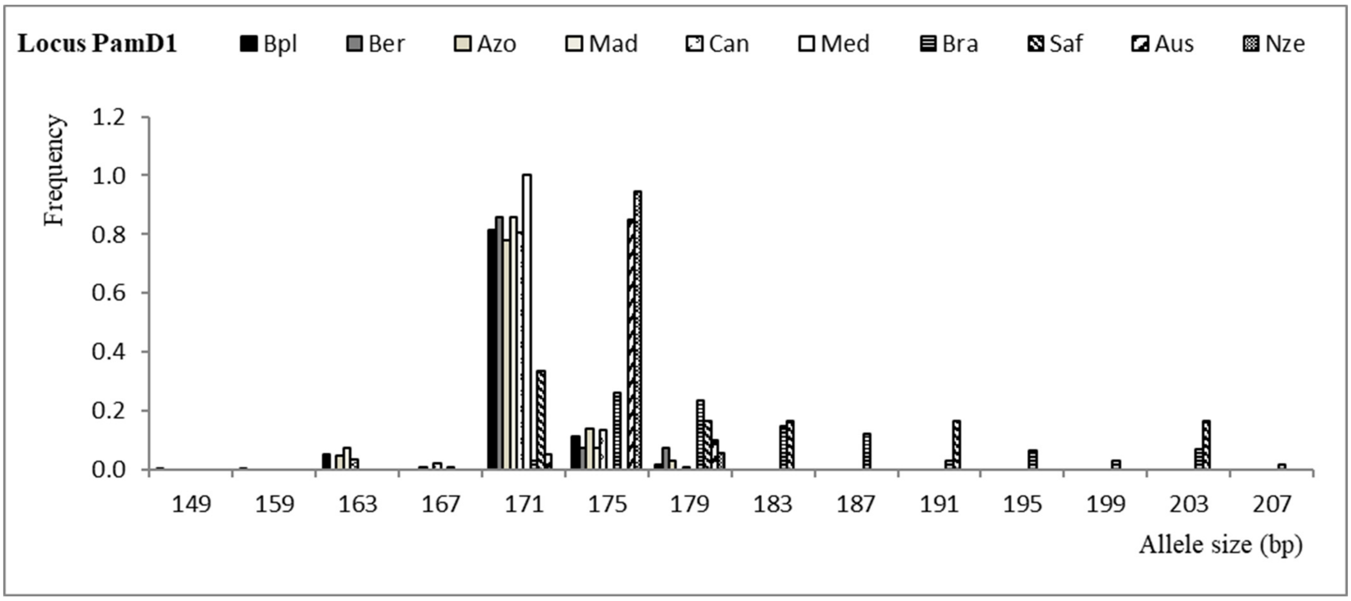

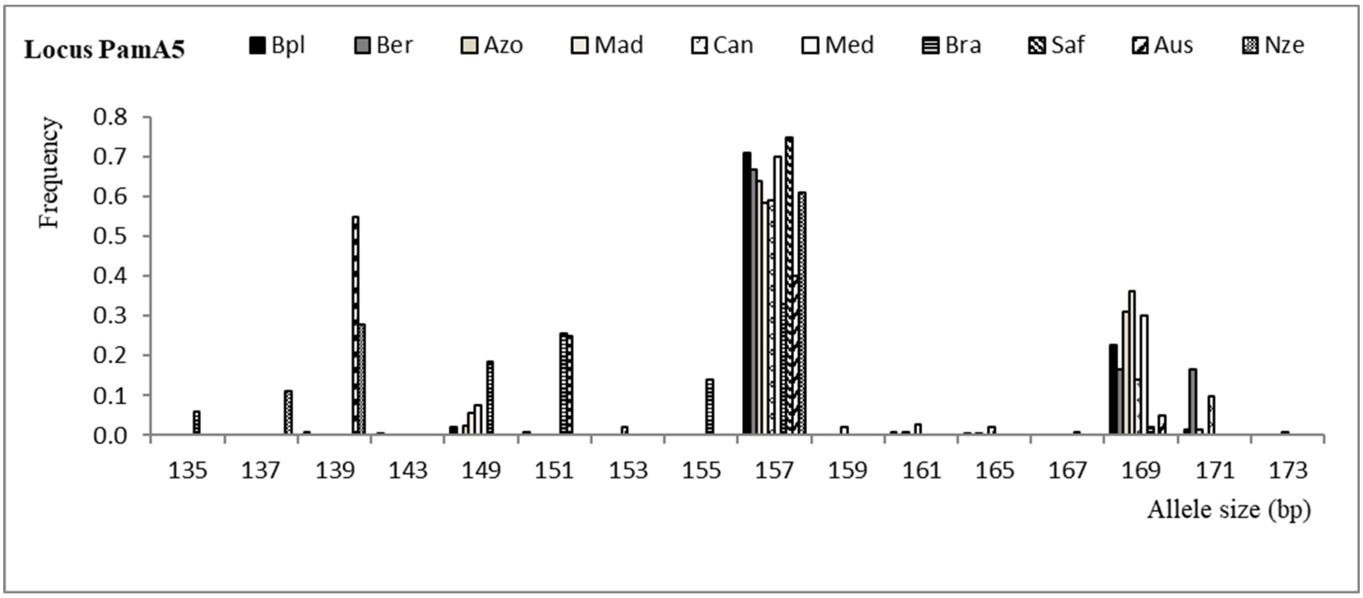

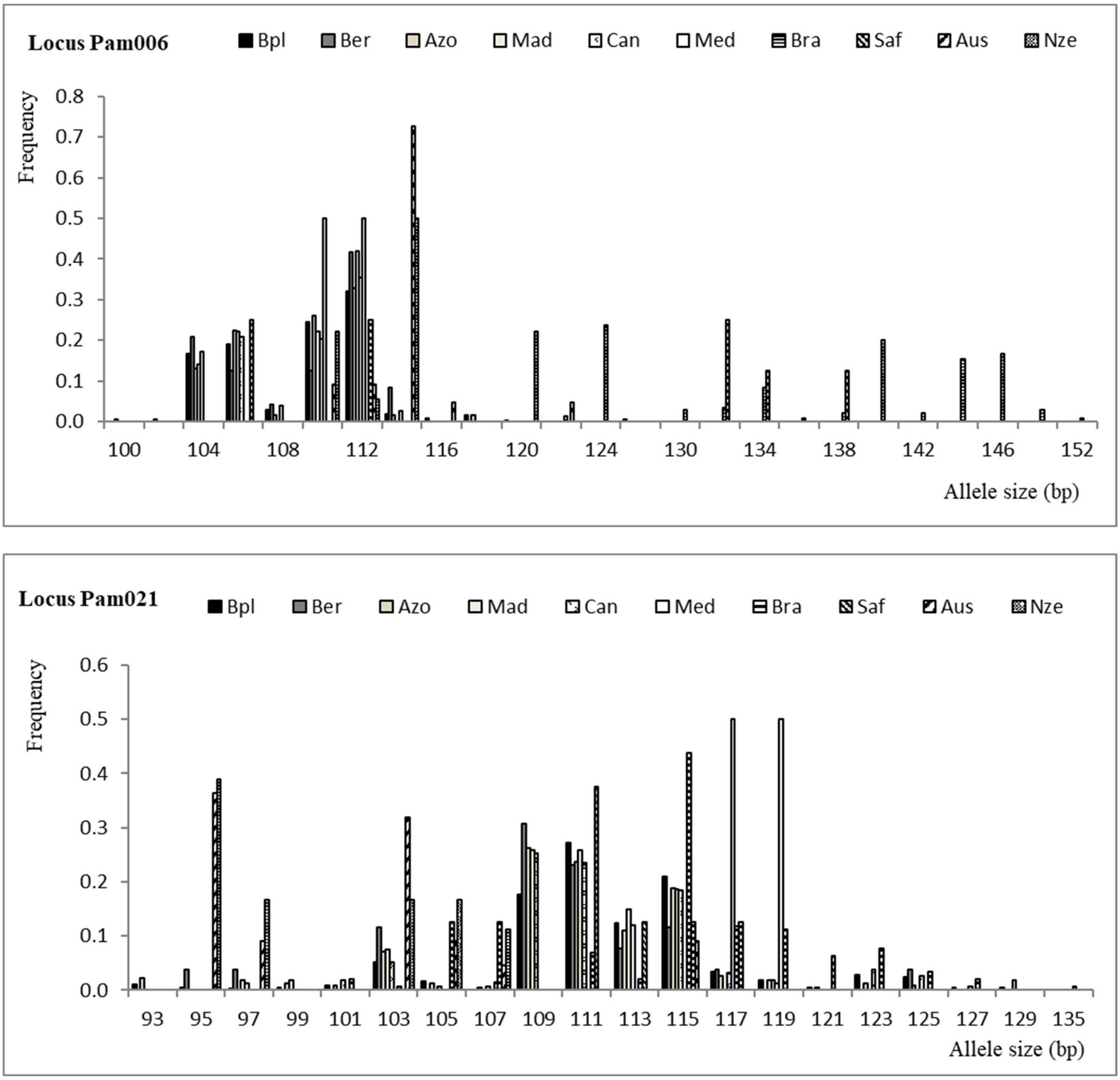

Figure S1. Distribution of allele frequencies (in bp) of four microsatellites in *Polyprion americanus* as broken per sampling region (sample codes in Table 1).

Table S1. Genetic parameters of four microsatellites in ten sampling areas of *Polyprion americanus* (sample codes in Table 1).

		Bpl	Ber	Azo	Mad	Can	Med	Bra	Saf	Aus	Nze
	N	140	7	123	21	74	4	71	6	10	9
	A	6	3	5	3	5	1	11	5	3	2
	Modal *A*	171	171	171	171	171	171	175–179	171	175	175
PamD1	Range *A*	149-179	171–179	163–179	163–175	163–179	171	167–207	171–203	171–179	175–179
	R_S	1.322	1.275	1.370	1.261	1.336	1.000	1.836	1.279	1.111	1.543
	H_E	0.322	0.274	0.371	0.265	0.337	0.000	0.837	0.850	0.289	0.111
	H_O	0.271	0.286	0.293	0.095	0.230	0.000	0.704	0.833	0.100	0.111
	F_{IS}	0.158	–0.043	0.210*	0.641	0.318	NA	0.159	0.020	0.654	0.000^{NA}
	Null	0.052	0.000	0.074	0.171	0.089	0.001	0.064	0.000	0.172	0.000
	N	141	6	118	18	72	5	68	4	10	9
	A	9	3	6	3	9	2	7	2	3	3
	Modal *A*	157	157	157	157	157	157	157	157	139	157
PamA5	Range *A*	139–171	157–171	149–171	149–169	149–173	157–169	135–169	151–157	139–169	137–157
	R_S	1.446	1.545	1.496	1.541	1.619	1.467	1.773	1.429	1.563	1.569
	H_E	0.446	0.567	0.496	0.539	0.620	0.450	0.774	0.417	0.561	0.556
	H_O	0.411	0.333	0.432	0.611	0.444	0.600	0.603	0.500	0.600	0.778
	F_{IS}	0.078*	0.412	0.129*	–0.133	0.284*	-0.333	0.221	–0.200	–0.069	–0.400
	Null	0.074	0.143	0.071	0.044	0.115	0.000	0.092	0.000	0.000	0.000
	N	190	12	123	25	79	2	72	4	11	9
	A	10	6	9	4	6	2	13	5	5	4
	Modal *A*	112	112	112	112	112	110–112	124–140	106–112–132	114	114
Pam006	Range *A*	104–126	104–114	100–118	104–112	104–114	110–112	122–152	106–138	110–122	110–120
	R_S	1.774	1.775	1.759	1.722	1.763	1.667	1.848	1.893	1.472	1.686
	H_E	0.774	0.780	0.759	0.726	0.763	1.000	0.849	0.875	0.473	0.694
	H_O	0.753	0.667	0.707	0.520	0.747	0.000	0.681	1.000	0.455	0.557
	F_{IS}	0.028	0.146	0.069	0.284	0.022	1.000	0.198*	–0.143	0.038	0.200
	Null	0.015	0.054	0.016	0.108	0.006	0.333	0.086	0.000	0.000	0.037
	N	191	13	114	27	79	1	72	4	11	9
	A	18	9	15	9	14	2	13	6	6	5
	Modal *A*	111	109–111	109–111	109–111	109–111	117–119	115	111	95–103	95
Pam021	Range *A*	93–129	95–125	93–125	97–129	97–127	117–119	101–135	105–117	95–115	95–107
	R_S	1.832	1.846	1.824	1.818	1.832	2.000	1.770	1.893	1.775	1.797
	H_E	0.832	0.856	0.824	0.818	0.832	NA	0.771	0.917	0.782	0.799
	H_O	0.744	0.615	0.763	0.815	0.734	1.000	0.625	0.750	0.636	0.778
	F_{IS}	0.107*	0.281	0.074	0.003	0.118	-1.000^{NA}	0.190*	0.182	0.186	0.026
	Null	0.039	0.101	0.030	0.000	0.047	0.000	0.033	0.000	0.110	0.000

Note: For each locus and sample is given: number of individuals (N), number of alleles (*A*), modal allele size (Modal *A*), allele range (Range *A*, in bp), allelic richness (*R*s), expected heterozygosity (H_E), observed heterozygosity (H_O), fixation index F_{IS} [83]. Significant departures from Hardy-Weinberg equilibrium were corrected with sequential test of Bonferroni (* $p \le 0.01$) [84] and null allele frequencies (*Null*).

Table S2. Migration rate M (± 95% CI) between wreckfish samples (codes in Table 1) from the first row (Donors) to those in the first column (Receptors) as inferred with BayesAss. Bolded figures indicate migration rates significantly different from zero. M-values on diagonal cells correspond to within-sample migration rates.

	Bpl	Ber	Azo	Mad	Can	Med	Bra	Saf	Aus	Nze
Bpl	0.850±0.092	0.002±0.003	**0.124±0.101**	0.002±0.003	0.013±0.021	0.002±0.003	0.002±0.003	0.002±0.003	0.002±0.004	0.002±0.003
Ber	0.096±0.091	0.682±0.028	0.066±0.085	0.015±0.028	0.067±0.081	0.015±0.027	0.015±0.028	0.015±0.028	0.015±0.028	0.015±0.027
Azo	**0.258±0.045**	0.003±0.005	0.714±0.048	0.002±0.005	0.011±0.016	0.002±0.005	0.002±0.004	0.003±0.005	0.003±0.005	0.002±0.005
Mad	**0.182±0.089**	0.009±0.017	0.072±0.085	0.675±0.017	0.017±0.029	0.009±0.016	0.009±0.016	0.009±0.016	0.010±0.019	0.009±0.017
Can	**0.153±0.093**	0.004±0.008	0.091±0.097	0.004±0.008	0.729±0.053	0.004±0.007	0.004±0.007	0.004±0.008	0.004±0.008	0.004±0.008
Med	**0.101±0.091**	0.021±0.039	0.048±0.074	0.021±0.039	0.032±0.055	0.689±0.041	0.023±0.042	0.022±0.040	0.021±0.039	0.021±0.038
Bra	0.011±0.015	0.004±0.008	0.008±0.013	0.004±0.008	0.004±0.008	0.004±0.008	0.952±0.025	0.004±0.008	0.005±0.009	0.004±0.007
Saf	0.041±0.061	0.025±0.047	0.033±0.056	0.024±0.044	0.039±0.063	0.023±0.042	0.077±0.073	0.692±0.046	0.023±0.043	0.023±0.042
Aus	0.019±0.033	0.015±0.028	0.023±0.041	0.015±0.028	0.016±0.031	0.015±0.029	0.018±0.033	0.015±0.027	0.849±0.073	0.015±0.029
Nze	0.018±0.033	0.018±0.033	0.018±0.033	0.018±0.032	0.018±0.033	0.018±0.033	0.018±0.032	0.018±0.033	**0.174±0.070**	0.685±0.034

Table S3. Migration rate M (±95% CI*)* between wreckfish samples (codes in Table 1) from the first row (Donors) to those in the first column (Receptors) as inferred with BIMr. Bolded figures indicate migration rates significantly different from zero. M-values on the diagonal correspond to within-sample migration rates.

	Bpl	Ber	Azo	Mad	Can	Med	Bra	Saf	Aus	Nze
Bpl	0.062±0.198	**0.200±0.128**	**0.186±0.171**	**0.243±0.104**	**0.182±0.132**	0.112±0.115	0.003±0.005	0.004±0.009	0.004±0.007	0.004±0.007
Ber	5.84E^{-11}±8.39E^{-10}	1.000±1.58E^{-6}	5.84E^{-11}±8.74E^{-10}	5.78E^{-11}±8.48E^{-10}	5.87E^{-11}±8.57E^{-10}	5.81E^{-11}±8.35E^{-10}	5.91E^{-11}±9.00E^{-10}	5.91E^{-11}±8.81E^{-10}	5.76E^{-11}±8.40E^{-10}	5.98E^{-11}±9.09E^{-10}
Azo	0.041±0.134	**0.159±0.147**	0.261±0.238	**0.283±0.142**	**0.155±0.117**	0.082±0.106	0.003±0.007	0.006±0.012	0.005±0.009	0.005±0.010
Mad	1.82E^{-10}±1.19E^{-9}	1.81E^{-10}±1.18E^{-9}	1.81E^{-10}±1.17E^{-9}	1.000±2.21E^{-6}	1.76E^{-10}±1.14E^{-9}	1.80E^{-10}±1.17E^{-9}	1.80E^{-10}±1.17E^{-9}	1.80E^{-10}±1.16E^{-9}	1.80E^{-10}±1.17E^{-9}	1.81E^{-10}±1.18E^{-9}
Can	2.96E^{-10}±3.48E^{-9}	2.96E^{-10}±3.42E^{-9}	2.86E^{-10}±3.24E^{-9}	2.92E^{-10}±3.33E^{-9}	1.000±error	2.93E^{-10}±3.39E^{-9}	2.90E^{-10}±3.37E^{-9}	2.99E^{-10}±3.54E^{-9}	3.01E^{-10}±3.59E^{-9}	2.98E^{-10}±3.49E^{-9}
Med	1.59E^{-10}±2.80E^{-10}	1.59E^{-10}±2.80E^{-10}	1.60E^{-10}±2.81E^{-10}	1.59E^{-10}±2.79E^{-10}	1.60E^{-10}±2.81E^{-10}	1.000±4.55E^{-6}	1.60E^{-10}±2.80E^{-10}	1.60E^{-10}±2.81E^{-10}	1.59E^{-10}±2.79E^{-10}	1.60E^{-10}±2.80E^{-10}
Bra	3.22E^{-10}±2.86E^{-9}	3.21E^{-10}±2.88E^{-9}	3.16E^{-10}±2.84E^{-9}	3.20E^{-10}±2.83E^{-9}	3.15E^{-10}±2.80E^{-9}	3.21E^{-10}±2.87E^{-9}	1.000±2.63E^{-6}	3.20E^{-10}±2.87E^{-9}	3.14E^{-10}±2.82E^{-9}	3.17E^{-10}±2.82E^{-9}
Saf	9.65E^{-11}±5.22E^{-10}	9.51E^{-11}±5.10E^{-10}	9.54E^{-11}±5.12E^{-10}	9.63E^{-11}±5.19E^{-10}	9.67E^{-11}±5.22E^{-10}	9.68E^{-11}±5.24E^{-10}	9.72E^{-11}±5.27E^{-10}	1.000±2.71E^{-6}	9.68E^{-11}±5.20E^{-10}	9.60E^{-11}±5.12E^{-10}
Aus	6.80E^{-11}±3.30E^{-10}	6.74E^{-11}±3.22E^{-10}	6.70E^{-11}±3.13E^{-10}	6.72E^{-11}±3.18E^{-10}	6.76E^{-11}±3.23E^{-10}	6.74E^{-11}±3.18E^{-10}	6.78E^{-11}±3.24E^{-10}	6.74E^{-11}±3.20E^{-10}	1.000±2.59E^{-6}	6.74E^{-11}±3.18E^{-10}
Nze	2.25E^{-10}±1.75E^{-9}	2.28E^{-10}±1.83E^{-9}	2.23E^{-10}±1.72E^{-9}	2.23E^{-10}±1.73E^{-9}	2.23E^{-10}±1.75E^{-9}	2.26E^{-10}±1.77E^{-9}	2.24E^{-10}±1.76E^{-9}	2.28E^{-10}±1.83E^{-9}	2.25E^{-10}±1.76E^{-9}	1.000±error
